# Naturally Lignan-Rich Foods: A Dietary Tool for Health Promotion?

**DOI:** 10.3390/molecules24050917

**Published:** 2019-03-06

**Authors:** Carmen Rodríguez-García, Cristina Sánchez-Quesada, Estefanía Toledo, Miguel Delgado-Rodríguez, José J. Gaforio

**Affiliations:** 1 Center for Advanced Studies in Olive Grove and Olive Oils, University of Jaen, Campus las Lagunillas s/n, 23071 Jaén, Spain; crgarcia@ujaen.es (C.R.-G.); csquesad@ujaen.es (C.S.-Q.); mdelgado@ujaen.es (M.D.-R.); 2 Department of Health Sciences, Faculty of Experimental Sciences, University of Jaén, 23071 Jaén, Spain; 3 Agri-food Campus of International Excellence (ceiA3), 14071 Córdoba, Spain; 4 Department of Preventive Medicine and Public Health, University of Navarra, 31008 Pamplona, Spain; etoledo@unav.es; 5 CIBER Fisiopatología de la Obesidad y Nutrición (CIBERObn), Instituto de Salud Carlos III, 28029 Madrid, Spain; 6 IdiSNA, Navarra Institute for Health Research, 31008 Pamplona, Spain; 7 CIBER Epidemiología y Salud Pública (CIBER-ESP), Instituto de Salud Carlos III, 28029 Madrid, Spain

**Keywords:** lignans, diet, antioxidants, health promotion, chronic diseases

## Abstract

Dietary guidelines universally advise adherence to plant-based diets. Plant-based foods confer considerable health benefits, partly attributable to their abundant micronutrient (e.g., polyphenol) content. Interest in polyphenols is largely focused on the contribution of their antioxidant activity to the prevention of various disorders, including cardiovascular disease and cancer. Polyphenols are classified into groups, such as stilbenes, flavonoids, phenolic acids, lignans and others. Lignans, which possess a steroid-like chemical structure and are defined as phytoestrogens, are of particular interest to researchers. Traditionally, health benefits attributed to lignans have included a lowered risk of heart disease, menopausal symptoms, osteoporosis and breast cancer. However, the intake of naturally lignan-rich foods varies with the type of diet. Consequently, based on the latest humans’ findings and gathered information on lignan-rich foods collected from Phenol Explorer database this review focuses on the potential health benefits attributable to the consumption of different diets containing naturally lignan-rich foods. Current evidence highlight the bioactive properties of lignans as human health-promoting molecules. Thus, dietary intake of lignan-rich foods could be a useful way to bolster the prevention of chronic illness, such as certain types of cancers and cardiovascular disease.

## 1. Introduction

Polyphenol-rich diets are suggested to possess health benefits. Polyphenols are micronutrients found in plants, and include flavonoids, stilbenes, phenolic acids, lignans and others [1]. They are secondary plant metabolites implicated in protection against pathogens and ultraviolet radiation [2]. Given their diverse chemical structures, different polyphenol classes likely possess differing health benefits [3]. It is therefore important to elucidate the specific potential benefits of each polyphenolic compound. Significant interest has been elicited by lignans, due to their steroid-analogous chemical structure. Accordingly, they are considered to be phytoestrogens. Lignans are bioactive compounds exhibiting various biological properties, including anti-inflammatory, antioxidant and antitumor activities [4]. Additionally, some epidemiological studies have proposed that lignans decrease the risk of cardiovascular disease, but their effects on other chronic diseases (e.g., breast cancer) remain controversial [5].

Lignans are found in relatively low concentrations in various seeds, grains, fruits and vegetables, and in higher concentrations in sesame and flax seeds [6]. Therefore, the level of lignan ingestion—and, thus, lignan bioavailability, depends on the type of diet consumed [7,8] and can be highly variable. The present review attempts to describe the potential beneficial effects of lignan intake on human chronic disease, depending on the dietary source.

## 2. Biosynthesis, Classification and Presence of Lignans in Foods

Lignans are a type of secondary plant metabolite exhibiting diverse structures [9]. Plants derive a complex array of secondary metabolites from only a handful of relatively simple propenyl phenols [10]. Biosynthesis of lignans is characterized by a remarkable increase in molecular complexity [10]. 

Lignans share common biosynthetic pathways, consist of two propyl-benzene units coupled by a β,β′-bond [11], and thus belong to the group of diphenolic compounds [12]. 

**Figure 1:**
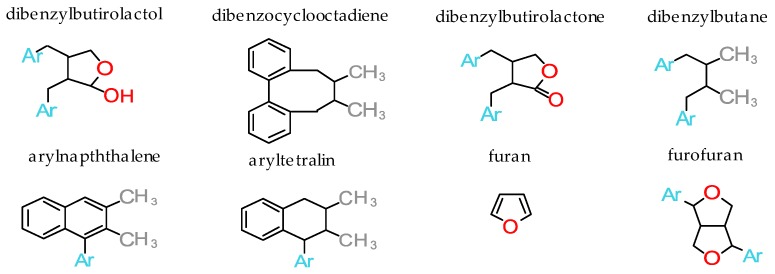
Structural subgroups of lignans (Ar=Aryl).

Lignans may be organized into eight structural subgroups (according to the manner in which oxygen is incorporated and the pattern of cyclization): Dibenzylbutyrolactol, dibenzocyclooctadiene, dibenzylbutyrolactone, dibenzylbutane, arylnaphthalene, aryltetralin, furan and furofuran (Figure 1). Each subgroup can be further subdivided according to lignan molecule oxidation level and identities of non-propyl aromatic rings present on side chains [13,14]. 

**Figure 2:**
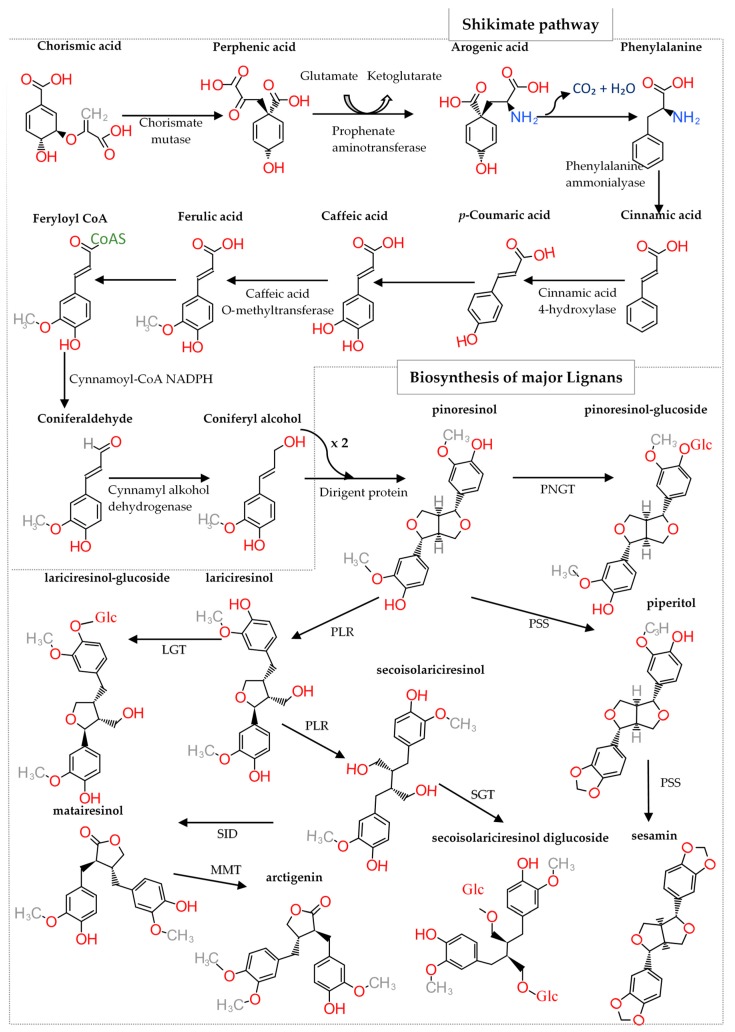
Biosynthetic pathway of lignans. NGT (pinoresinol glucosyltransferase), PSS (piperitol/sesamin synthase), PLR (pinoresinol/lariciresinol reductase), LGT (lariciresinol glycosyltransferase), SGT (secoisolariciresinol glycosyltransferase), SID (matairesinol O-methyltransferase), MMT (matairesinol O-methyltransferase), Glc (Glucoside).

Of the eight lignan subclasses, synthesis of furofurans—which exhibit a 2,6-diaryl-3,7-dioxabicyclooctane skeleton—is initiated by the enantioselective dimerization of two coniferyl alcohol units derived from the shikimate biosynthetic pathway (Figure 2) [14]. To date, 53 species of furofuran lignans have been reported in 41 genera of 27 plant families, including Thymelaeaceae, Styracaceae, Scrophulariaceae, Saururaceae, Rutaceae, Rhizophoraceae, Piperaceae, Pedaliaceae, Orobanchaceae, Myristicaceae, Magnoliaceae, Lauraceae, Lamiaceae, Geraniaceae, Dioscoreaceae, Cyperaceae, Cupressaceae, Compositae, Combretaceae, Cactaceae, Aristolochiaceae, Arecaceae, Araliaceae, Aquifoliaceae, Apocynaceae, Acoraceae and Acanthaceae. Furofuran lignans are present in the bark, bulbs, leaves, seeds, stems and roots of these plants [14]. 

However, depending on the enzyme that catalyzes modification of the precursor metabolite, a variety of lignans can be synthesized (Figure 2). The major lignans—which possess numerous pharmacological properties—are artigenin, enterodiol, enterolactone, sesamin, syringaresinol, medioresinol, (−)-matairesinol, (−)-secoisolariciresinol, (+)-lariciresinol and (+)-pinoresinol, among others [15].

Currently, there is a growing interest in the presence of lignans in foodstuffs, given the potentially beneficial bioactive properties of the former (anti-estrogenic, antioxidant and anti-carcinogenic activities) [16]. The chief sources of dietary lignans are various vegetables and fruits, legumes, whole grain cereals and oilseeds [16,17]. Among edible plant components, the most concentrated lignan sources are sesame and flax seeds (Table 1 and Table 2) [6]. Specifically, flax seeds contain approximately 294.21 mg/100 g lignan, at present the maximal known content of any foodstuff. Sesame seeds exhibit the second-highest lignan concentration, with sesaminol as the major constituent, at 538.08 mg/100 g [6]. Flaxseed and cashew nuts are also relatively rich in lignans (containing 257.6 and 56.33 mg/100 g, respectively) [6].

Regarding cereal grains (Table 3), lignans are largely concentrated in their outer layers [19,20]. In cereal grains, the highest lignan concentration is found in the fiber-rich outer layers (seed coat and pericarp), as well as the aleurone layer, whereas the lowest concentration is found in the inner endosperm [21,22]. 

Ordering species by lignan content produces the following list: Dhurra < brown rice < red rice < quinoa < millet < corn < amaranth < barley < buckwheat < wild rice < Japanese rice < spelt < oat < triticale < wheat < rye [6]. Regarding vegetables (Table 4), the brassica family may contain between 185 and 2.321 mg /100 g of lignan, mainly pinoresinol. Peppers, French beans, carrots and courgettes also exhibit a relatively high lignan content, ranging from 0.113 to 0.273 mg/100 g. Other foods, such as spinach, white potatoes and mushrooms—contain below 0.1 mg/100 g of lignan. Fruits exhibit a lower lignan content than seeds or vegetables (Table 5 and Table 6), ranging from 11.57 mg/100 g for apricots to 0 mg/100 g for banana, with green grapes and kiwi fruit falling somewhere between these extremes [6].

The highest lignan content is observed in non-alcoholic beverages, such as tea (0.0392–0.0771 mg/100 g), which also contains other polyphenols (Table 7). Coffee is another important source of lignans, although concentration varies by type of coffee, ranging from 0.0187 to 0.0313 mg/100 g. Regarding alcoholic beverages, red wine contains an average of 0.080 mg/100 mL, whereas white wine contains only approximately 0.022 mg/100 g [23].

Furthermore, the chief source of dietary fat in Mediterranean countries—extra virgin olive oil (EVOO)—has garnered much interest regarding its beneficial properties, largely attributable to its polyphenol profile (Table 8). Lignans are the second most abundant polyphenolic class present in EVOO; of these, the most abundant across different EVOO types are pinoresinol (1.17–4.12 mg/ 100 g) and 1-acetoxypinoresinol (0.27–6.69 mg/ 100 g) [7,24,25].

Thus, given the presence of lignan in many common foodstuffs and beverages, its intake occurs frequently, on a near-daily basis. For example, in a Dutch population, the major dietary sources of lignan were fruits (7%), bread (9%), seeds and nuts (14%), vegetables (24%), and beverages (37%) [6]. Similarly, in a cohort of French women, the major dietary sources of lignan were vegetables and fruits (0.2% from legumes, 0.6% from potatoes, 30% from vegetables, and 35% from fruits), followed by alcoholic beverages (5%), coffee (5%), cereals (7%) and tea (11%) [6,26,27]. 

## 3. Bioavailability

**Figure 3:**
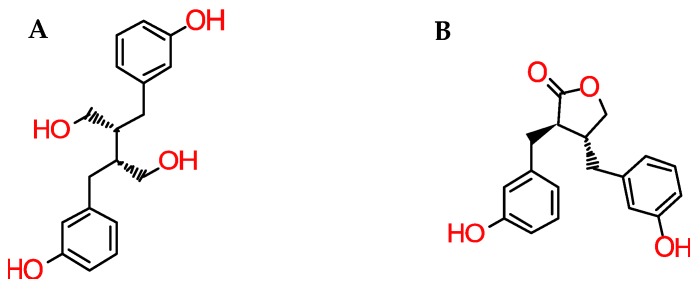
Chemical structure of enterodiol (**A**) and enterolactone (**B**).

Only a handful of studies exist regarding post-consumption lignan bioavailability, including only very limited human pharmacokinetic studies. After ingestion, plant lignans are metabolized by intestinal bacteria, undergoing transformation to mammalian lignans (enterolactones and enterodiols (Figure 3)) prior to absorption [16,28]. This apparently considerably decreases the risk of diverse types of cancer, particularly of the colon, prostate and breast [16,29]. 

Many studies demonstrate a positive correlation between plant lignan intake and plasma enterolignan levels [30]. After lignan ingestion, enterolactone and enterodiol are the first lignans to become detectable in human biological fluids [28]. The half-lives of these compounds in plasma are approximately 13 and 5 h, respectively [31], and they remain detectable even up to 8–10 h after plant lignan consumption [32]. Furthermore, their intestinal metabolism into mammalian forms appears indispensable for colonic absorption, and the colonic barrier is capable of conjugating enterolignans [28,33].

The concentration of enterodiol and enterolactone in biological fluids varies significantly by geographic region [28]. A study examining mammalian lignan pharmacokinetics in both men and women after lignan solution intake found that enterodiol and enterolactone, respectively, exhibit absorption half-lives of 3.4 and 8.4 h, reach maximum plasma concentrations of 65 and 42 mmol/L [28], exhibit elimination half-lives of 4.6 and 15.1 h, and exhibit maximum retention times of 23.9 and 43.2 h [28,34]. Thus, while enterolactone is more rapidly absorbed than enterodiol, the former attains a lower maximum plasma concentration [28]. 

During lignan metabolism, the initial (cytochrome P450-mediated) step involves conjugation to glucuronic acid and sulfate, followed by enterohepatic recirculation [35]. Chaojie et al. (2013) that glucuronidation of flax seed lignans significantly involves liver and intestinal microsomes [36]. Some studies demonstrate that flax seed-derived lignan metabolites distribute mainly to the intestine (largely to the caecum), kidneys, uterus, prostate and liver [37]. Of these locations, the highest concentration of lignan metabolites is observed in the liver [37].

Human breast cyst, prostatic, and seminal fluid (as well as prostate tissue) lignan concentration has been determined [38,39]. As in circulation, the common mammary form of lignan is enterolignan, while urinary forms are essentially monoglucuronides [28]. Furthermore, inter-individual variations in gut microbiota and hepatic enzymes may modulate mammalian lignan metabolism and bioactivity [33]. 

Moreover, lignan bioavailability also depends on diet. For example, diets rich in flax seed increase production of gut microbiota-derived enterolignans in a murine model, and lead to high tissue and plasma concentrations of sulfate and glucuronide conjugates (the major flax-derived lignan metabolites) [8,40]. 

Other studies have demonstrated that plant lignans, such as sesamin are quickly absorbed, apparently from the small intestine and become detectable in systemic circulation within a few hours after ingestion [22,41]. For example, lignans have been observed in porcine plasma 3 h after cereal intake [42]. On the one hand, it has been empirically demonstrated that plant lignans are rapidly absorbed from the small intestine after intake of a diet rich in cereals [22]. On the other hand, various factors—e.g., the use of oral antibiotics and inter-individual variations in gut microflora, as well as diet—impact lignan pharmacokinetics [43]. For example, seed maturation state can alter oral lignan bioavailability [44]. 

## 4. Lignan Content of Various Regional Diets 

Dietary lignan consumption varies mainly with geographic location, but diet patterns are also subject to cultural and ethnic group influences.

### 4.1. Mediterranean Diet

The traditional Mediterranean diet is predominantly plant-based, characterized by a low intake of sweets; low meat products and red meat; a moderate intake of fish, poultry and fermented dairy products; a high intake of unprocessed cereals, legumes, nuts, fruits and vegetables [45]; the use of EVOO as the principal source of added fat; and moderate consumption of red wine [45]. Health benefits of this diet are essentially attributable to increased consumption of fiber and bioactive compounds (including antioxidants and functional fatty acids and lipids), as well as to a low intake of saturated fats [45,46]. 

Lignan sources in the diet of a Mediterranean population included garlic, onions, vegetables, including leafy greens, grains and seasonal fruits, including citrus, with each accounting for diverse proportions (11–70%) and subtypes of total polyphenols consumed [47]. 

Indeed, many typical Mediterranean diet foods (e.g., cereals) exhibit a high concentration of both lignans and other phenolic compounds [48]. 

Recently, the role of whole grain cereal intake in chronic disease prevention has been evaluated. Numerous studies propose a connection between lignan intake—as part of a wholegrain-based diet—and decreased incidence of chronic diseases, including cardiovascular disease, cancer and diabetes [5]. 

Thus, the major dietary lignan sources in the Mediterranean diet are vegetables and fruits, legumes, wholegrain cereals and oilseeds [3]. Additionally, another component of the Mediterranean diet, the chestnut, represents an excellent source of calcium, antioxidants and phenolic compounds [16,49]. Furthermore, EVOO consumption is an essential part of the Mediterranean diet. In fact, regular EVOO consumption is associated with a lower incidence of atherosclerosis, cardiovascular disease and some types of cancer [50,51,52]. This effect may be attributable to the high concentrations of (+)-1-acetoxypinoresinol and (+)-pinoresinol present in EVOO [53,54].

### 4.2. Northern Hemisphere Diet 

This diet is observed in Northern and Nordic European regions, and is characterized by a high level of consumption of seaweed, shellfish, fatty fish (such as mackerel, herring and salmon), lean meats, rapeseed oil, legumes, nuts (such as almonds), vegetables, fruits (such as berries), whole grains (such as oats), low-fat dairy, and restricted salt and sugar intake [55,56]. In Nordic countries, the major dietary sources of plant lignans are vegetables, fruits and wholegrain cereals [57]. 

Among the many frequently-consumed plant species exhibiting a high lignan content, some species occur mainly in the Northern Hemisphere (e.g., *Cirsium spp*. of the family Asteraceae) [58]. The vegetative structures of these plants contain triterpenes, polyacetylenes, phenolic acids, flavonoids and alkaloids [58]. The most recent phytochemical studies of European *Cirsium spp*. demonstrate that their seeds are rich sources of neolignans and lignans [58,59]. 

### 4.3. Indian Diet

Various categories of food products make up a significant portion of the typical Indian diet, including fish, grapes, chocolate, oils, coffee, tea, biscuits and bread [60]. 

The fruit of *Morinda citrifolia* (Indian mulberry) has been extensively traditionally utilized in the treatment of cancer, diabetes, high blood pressure, diarrhea, headache and inflammation, largely due to its high lignan content [61,62].

Sesame is a typical component of the Indian diet, and both sesame seeds and oil are rich in lignans [63]. Sesame oil is recognized for both its notable resistance to oxidation and its nutritional value [64,65,66]. Despite lignans comprising only a small proportion (0.5 to 1.0%) of total sesame seed mass, the main sesame lignans—such as (+)-sesaminol, (+)-sesamolin and (+)-sesamin glucosides—have garnered attention for their notable health-promoting properties (demonstrated both in vitro and in vivo), including anti-inflammatory, antioxidant and anti-hypertensive activities [63].

Long-term intake of (+)-sesaminol has been proposed to inhibit the pathogenic extracellular β-amyloid aggregation observed in Alzheimer’s Disease [67]. Similarly, (+)-sesamin exhibits protective activity against prostate and breast cancers [68], and is a precursor to enterodiol and enterolactone (which have been shown to possess anti-cancer, antidiabetic and anti-ageing properties [64]).

### 4.4. Asian Diet

The Asian diet is characterized by an elevated consumption of rice, noodles, spices and vegetables, sesame seeds and oil [69]. Additionally, seafood, tofu and other soy products are commonly consumed [70]. Many major plant sources of lignans occur in Asia; these are habitually included in the diet, and in China are also used as medicinal plants. Such plants include *Articum lappa*, whose fruit extracts and seeds are a rich source of bioactive lignans [70], including arctiin and arctigenin. These two lignans exhibit anti-inflammatory activities (e.g., inhibition of lipopolysaccharide-induced nitric oxide production and release of pro-inflammatory cytokines in murine macrophages in vivo) [70,71]. In addition, when tested on diverse cancer cell lines, arctigenin possesses potent apoptotic and anti-proliferative activities [70,72].

Certain medicinal herbs are usually used as an aqueous infusion. Among them, *Isodon spp*. and *Tripterygium spp*.

The genus Isodon comprises nearly 150 species found in the subtropical and tropical regions of Asia and represents an excellent lignan source [73]. Some species, such as *Isodon japonica*, have been used in traditional Chinese medicine to treat (for example) arthralgia, stomach-ache, mastitis, gastritis and hepatitis [73,74]. *Isodon rubescens* has also been used in traditional medicine for its hypotensive, antioxidant, immunological, antimicrobial, antitumor and anti-inflammatory properties [73].

*Tripterygium wilfordii Hook f.*, a traditional medicinal herb, may ameliorate symptoms of rheumatoid arthritis and other autoimmune diseases [75]. Several phytochemical research studies have isolated hundreds of bioactive compounds—including lignans—from the root of this plant [75,76].

Chinese traditional medicine has long made use of *Schisandra chinensis Baill*. fruit as a sedative and antitussive tonic [77]. This fruit is additionally used in other countries in the production of functional foods, jam and beverages. Dibenzocyclooctadiene lignans isolated from *S. chinensis* exhibit anti-inflammatory and antioxidant properties, as well as improving cognitive functions (e.g., memory) [77]. In addition, prior studies have reported that *S. chinensis* fruit extracts—in which the major bioactive constituents are lignans—exert a neuroprotective effect and possess bioactivity which may help prevent Alzheimer’s Disease [78]. Furthermore, *S. chinensis* fruit may have positive effects on the liver, as well as on the gastrointestinal, immune, sympathetic and central nervous systems [79,80]. Lignan extracts have been shown to successfully suppress hepatocellular carcinoma cell proliferation and to prevent chemical toxin-induced hepatic injury [79]. However, only 2% of the total *S. chinensis* fruit is made up of lignans, and most of these are present in the seeds, which are usually removed during manufacture of fruit-derived products [79].

The *Schisandra glaucescens Diels* vine is extensively distributed across the Southeastern Sichuan and Western Hubei regions of China [81]. The stem of this vine has been used as an analgesic in diverse conditions, including arthritis, rheumatism, and contusions. As yet, one sesquiterpenoid, 25 lignans and 43 triterpenoids have been isolated from *S. glaucescens* [81]. In addition, *S. glaucescens* berries are thought to exert beneficial effects on the kidneys and lungs, relieving the symptoms of asthma for example [82].

*Crataegus pinnatifida* has been employed by the functional foods industry. Some studies have reported that it has the ability to protect against low-density lipoprotein (LDL) oxidation, to scavenge free radicals, and to exert an anti-inflammatory effect [83,84]. *C. pinnatifida* is mostly consumed as fresh fruit, processed juice or jam. Juice and jam manufacture results in a significant quantity of by-products, including seeds and leaves [84].

*Schisandra sphenanthera* is mainly located in Southwest China. A diversity of triterpenoids and lignans has been isolated from its leaves, stems, and fruit [85].

The roots, stems, fruit, and leaves of *Kadsura coccinea* are used medicinally, and its fruit, particularly, exhibits significant medicinal and nutritional properties [86]. Its bioactive triterpenoids and lignans have garnered interest for their reported bioactivities, including anti-inflammatory and anti-tumor effects [86,87,88].

*Zanthoxylum schinifolium* has been employed to stimulate blood circulation, as well as in the treatment of various diseases [89,90]. Due to its exceptional taste and characteristic aroma (usually described as green, spicy, floral, and fresh), *Z. schinifolium* fruit is used as a spice in many traditional Asiatic cuisines [89]. Prior pharmacological studies have demonstrated that the leaves and fruit of this plant possess medicinal properties, including antitumor, anti-inflammatory, and antioxidant activities, as well as inhibition of both platelet aggregation and monoamine oxidase production [89,91].

### 4.5. Latin-American Diet

The basis of the Latin-American diet consists of maize (corn), potatoes, peanuts and beans. This diet also includes flax seed. As mentioned above, *Linum usitatissimum L.* (flax seed) represents one of the best dietary sources of lignans, exhibiting a higher lignan content than legumes or grains [8]. Diets rich in flax seed are associated with a reduced risk of various diseases, including cardiovascular disease, osteoporosis, diabetes, and prostate and breast cancers [8,92]. Likely mechanisms include the ability to decrease circulating glucose, LDL and total cholesterol levels [93,94]. Furthermore, *L. usitatissimum* has significant commercial applications, in the manufacture of linen fiber for example [94]. In terms of lignans, flax seed contains mainly secoisolariciresinol and secoisolariciresinol diglucoside, but matairesinol is also present in small quantities [95]. Indeed, >95% of total flax seed mass consists of secoisolariciresinol diglucoside, which is predominantly localized in the seed’s fibrous hull [96] rather than its interior [97].

Asian diet appears to facilitate the highest intake of lignans, in forms which also result in higher bioavailability. This is due largely to a high level of vegetable consumption, as well as the use of lignan-rich plant infusions in traditional medicine.

## 5. Human Studies Concerning Lignan Bioactivity

Recently, interest in identifying new sources of health-promoting natural compounds has increased. However, there are few human epidemiological studies that evaluate lignans bioactivity. Laboratory research, carried out on cell and animal models, concluded that lignans possess antimicrobial, anti-inflammatory and anti-oxidant activities, among others.

About antimicrobial activity, various lignans have exhibited antiviral and antibacterial activity, e.g., against Gram-positive bacteria through alteration of biofilm formation, bacteria metabolites, membrane receptors and ion channels [98]. For instance, pinoresinol has demonstrated activity against some virus [99].

Concerning anti-inflammatory activity, some lignans have the capacity to inhibit NF-kB activity (transcription factor involves on the expression of inflammatory cytokines) on human mast cells (HMC-1). Thus, reduced pro-inflammatory cytokines production. Furthermore, lignans are able to suppress nitric oxide (NO) generation and decrease inflammatory cell infiltration [100,101,102].

Regarding anti-oxidant activity, various bioactive natural compounds—including phenols from grains, vegetables and fruits—are rich dietary sources of phytochemicals and vitamins, both of which guard against oxidative stress [84,103]. A free radical formation is an inevitable byproduct of cellular metabolism, and cells also *require* a certain level of reactive oxygen species (ROS) to carry out a normal cellular process [70]. Nevertheless, accumulation and/or overproduction of ROS can damage cellular constituents, including DNA [70], and play an important role in the pathogenesis of various severe disorders, including chronic inflammation, cancer, neurodegeneration and atherogenesis [84].

Many studies have demonstrated the strong antioxidant activity of plant extracts, attributable to several highly-effective antioxidants, including lignans (e.g., lariciresinol, matairesinol, secoisolariciresinol, pinoresinol, and nortrachelogenin) [104]. Among the natural antioxidants, lignans exhibit particularly high antioxidant efficiency and thus have potential as preventive and/or therapeutic clinical tools [105].

In recent years, a significant effort has been devoted to analyzing the lignan consumption of various populations (Table 9). Most studies have focused on post-menopausal women, due to lignans being phytoestrogens that ameliorate menopausal symptoms and consequences (e.g., climacteric symptoms, osteoporosis and estrogen-dependent cancers) [106].

### 5.1. Cancer

Various cohort studies have investigated dietary lignan anticancer bioactivity. As McCann et al. (2010) describe in the “Western New York Exposures and Breast Cancer” study, lignan intake among post-menopausal women with breast cancer significantly reduced the risk of mortality from breast cancer (Hazard Ratio (HR) 0.29, 95% Confidence interval (CI) 0.11–0.76), as well as significantly reducing the risk of all-cause mortality (HR 0.49, 95% CI 0.26–0.91) [107]. Other research based on the Swedish Mammography Cohort (SMC) also detected a statistically significant inverse association between breast cancer risk and lignan consumption among post-menopausal breast cancer patients [108]. Interestingly, the “Ontario Women’s Diet and Health Study” reported that neither lignan nor isoflavone consumption by a Canadian cohort correlated with a significant reduction in breast cancer risk [109]. Nonetheless, some studies do propose that isoflavone consumption correlates with a minor reduction in breast cancer risk in both pre- and post-menopausal women [109,110]. In addition, a cohort study examining the association between flax seed and flax bread intake and breast cancer risk demonstrated that flax seed intake was associated with a significant reduction in breast cancer risk (Odds Ratio (OR) 0.82, 95% CI 0.69–0.97) [111]. Furthermore, Buck et al. (2011) demonstrated that high serum enterolactone levels in post-menopausal breast cancer patients are associated with improved overall survival rates [109,112].

Another study, based on data from the United States Cancer Center Support Grant, investigated the association between individual breast cancer estrogen receptor (ER) status and lignan intake [113]. Higher lignan consumption was inversely correlated with the risk of ER^−^ breast cancer among premenopausal women (OR 0.16, 95% CI 0.03–0.44) and with the risk of ER^+^ breast cancer among post-menopausal women (OR 0.64, 95% CI 0.42–1.00) [113]. Although this effect was largely independent of specific lignan class, it predominantly correlated with matairesinol and lariciresinol intake levels [113]. In addition, this study examined associations between breast tumor subtype and dietary lignan intake, demonstrating that a reduction in premenopausal triple-negative (HER2^−^PR^−^ER^−^) breast cancer risk (OR 0.16, 95% CI 0.04–0.62) was associated with higher lariciresinol and pinoresinol intake [113]. This finding agrees with that of a German case-control study that demonstrated a correlation between high intake of pumpkin and sunflower seeds (rich sources of lariciresinol and pinoresinol) and a statistically significant reduction in post-menopausal ER^+^ breast cancer risk (OR = 0.88, 95% CI = 0.77–0.99, p for trend = 0.02) [109,114].

Two recent meta-analyses have corroborated that high levels of plant lignan consumption correlate with a modest reduction in post-menopausal breast cancer risk (13 studies; Risk Estimated (RE) 0.86, 95% CI 0.78–0.94) [115,116].

Dietary lignan intake is also associated with a reduced risk for other cancer types (e.g., esophageal and gastric adenocarcinoma, as well as colon cancer), but very few human studies have been conducted.

A Swedish study indicates that dietary lignan intake correlates with decreased risk of gastroesophageal junction adenocarcinoma [117]. However, another Swedish study examining the Swedish Cancer Registry database did not find a clear association between dietary lignan consumption and development of gastric or esophageal adenocarcinoma [118]. Yet another (case-control) study indicated that a diet rich in resveratrol, quercetin and lignans (characterized by low intake of milk, but high intake of wholegrain bread, vegetables, wine and tea) may decrease the risk of developing such cancers [103].

Regarding colorectal cancer, Zamora-Ros et al. (2015) evaluated the association of lignan and flavonoid consumption with overall survival time and risk of recurrence in Barcelona (Spain) [119]. After a mean of 8.6 years’ follow-up, 77 of the 319 (24.1%) patients in the cohort had experienced recurrence (excluding cases with metastasis that could not be resected), 133 of 409 (32.5%) patients had died, and no association was noted between consumption of any flavonoid subclass or total lignans and colorectal cancer risk [119].

Concerning prostate cancer risk, it has been studied its association with plasma enterolactone concentrations. Wallström et al. (2018) evaluated a population of Swedish men with 1010 cases and 1817 controls. After a mean follow-up of 14.6 years; there were no significant associations between the incidence of prostate cancer and plasma enterolactone (OR 0.99, 95% CI 0.77–1.280) [120]. Other study carried out at Danish men, neither found an association between prostate cancer mortality and plasma enterolactone [121]. However, two other pieces of research on humans, from 2003 and 2006, obtained positive results based on dietary phytoestrogen intake [122,123]. A Swedish case-control study indicated that lower prostate cancer risk is related to certain phytoestrogen-rich foods [123].

Given such mixed results, additional studies examining the effect of human lignan intake on cancer risk are necessary. Specifically, most existing studies have not examined the relevance of the specific dietary lignan source.

### 5.2. Cardiovascular Disease

Neolignans and flax lignans are reportedly relevant in diabetes, hypercholesterolemia and cardiovascular disorders [124]. In addition, the anti-aging role of lignans has recently been described [125]. Such lignan characteristics may be relevant to the reduction of cardiovascular disease risk in post-menopausal women. Indeed, an inverse association exists between high lignan consumption and the development of hypertension and cardiovascular disease [126]. Furthermore, prospective and cross-sectional epidemiological evidence suggests that dietary lignan intake reduces cardiovascular disease risk in post-menopausal women and elderly men by modifying traditional risk factors [127].

Jacobs et al. (2000) demonstrated that the risk of mortality is inversely associated with whole grain consumption in post-menopausal women [128]. Another study described how four weeks’ consumption of a whole grain cereal-rich diet exerted a reasonable cholesterol-lowering effect in healthy post-menopausal women [17].

However, a Warsaw population-based cross-sectional study conducted by the National Institute of Cardiology demonstrated that total dietary lignan consumption does not correlate with the occurrence of cardiovascular diseases, nor with cardiovascular risk factors (including central obesity, hypercholesterolemia and hypertension) in post-menopausal women [126]. Nevertheless, this study attributed a potentially-beneficial effect of lignan intake on hypercholesterolemia specifically to lariciresinol [126].

In a Finnish population, the highest serum enterolactone concentrations correlated with a lower risk of all-cause mortality, including from cardiovascular disease [129]. Enterolactone is a metabolite of lariciresinol, pinoresinol, secoisolariciresinol and matairesinol, and very low matairesinol intake does demonstrate an inverse relationship with endothelial dysfunction and vascular inflammation [127].

### 5.3. Other Diseases

Most studies have focused on the effects of lignan-rich food consumption in the prevention of cancer and cardiovascular disease. However, some observational studies have investigated the relationship between regular consumption of plant lignans and the risk of developing other lifestyle-related diseases. A study based on the European Prospective Investigation into Cancer and Nutrition cohort proposed that improved cognitive performance in post-menopausal women is associated with higher dietary phytoestrogen consumption (predominantly lignans in Western diets) [130]. Thus, it has been suggested that low-grade chronic inflammation contributes to the prevalence of chronic lifestyle-related diseases. The relationship between lignan consumption and inflammatory markers (e.g., C-reactive protein (CRP)) was studied in a United States cohort, demonstrating that a beneficial inflammatory marker profile is associated with adult lignan consumption [131].

## 6. Conclusions

Taken together, reviewed data support the recently increased interest in lignan health-promoting properties. Due to their various bioactive properties, dietary intake of lignan-rich foods may prevent certain types of cancers (e.g., breast cancer in post-menopausal women and colon cancer). Regarding chronic lifestyle-related diseases, some pieces of evidence indicate that lignan intake is associated with a lower risk of developing cardiovascular disease. Nonetheless, further human studies are warranted to evaluate lignan bioavailability resulting from different traditional dietary patterns, in order to influence the rational promotion of healthy lignan-rich diets.

## Figures and Tables

**Table 1:** Lignan content of sesame seed (mg/100g food). Data collected from phenol explorer [18].

**Seeds**	**HMA**	**HSE**	**OXO**	**ARC**	**CYC**	**CON**	**DIM**
Sesame seed	7.2	0.01	0.7	0.01	1.77	0.75	0.39
	**ISO**	**LAR**	**LAS**	**MAT**	**MED**	**NOR**	**SEC**
	1.61	10.37	0.08	29.79	4.15	0.08	0.1
	**SECS**	**SES**	**SEI**	**SEN**	**SYR**	**TOD**	**Total**
	0.01	538.08	102.86	133.94	0.2	2.47	834.57

Lignans: 7-Hydroxymatairesinol (HMA), 7-Hydroxysecoisolariciresinol (HSE), 7-Oxomatairesinol (OXO), Arctigenin (ARC), Conidendrin (CON), Cyclolariciresinol (CYC), Dimethylmatairesinol (DIM), Isohydroxymatairesinol (IHM), Isolariciresinol (ISO), Lariciresinol (LAR), Lariciresinol-sesquilignan (LAS), Matairesinol (MAT), Medioresinol (MED), Nortrachelogenin (NOR), Secoisolariciresinol (SEC), Secoisolariciresinol-sesquilignan (SECS), Sesamin (SES), Sesaminol (SEI), Sesamolin (SEN), Syringaresinol (SYR), Todolactol A (TOD).

**Table 2:** Lignan content of seeds (mg/100g food) [18].

	LAR	MAT	MED	SEC	SYR	Total
Other Seeds						
Flaxseed	11.46	6.68	-	257.6	-	257.6
Sunflower seed	0.67	0.67	-	0.18	-	1.52
Nuts						
Almond	0.03	3 × 10^−4^	-	0.07	-	0.10
Brazil nut	-	0.01	-	0.77	-	0.78
Cashew nut	49.6	2.5 × 10^−3^	-	6.73	-	56.33
Chesnut	7.8 × 10^−3^	8.42 × 10^−3^	-	0.2	-	0.21
Hazelnut	0.01	3.3 × 10^−3^	-	0.05	-	0.06
Peanut	4.1	2.5 × 10^−3^	-	2.7	-	6.8
Pecan nut	8.4 × 10^−3^	3.15 × 10^−3^	-	0.01	-	0.02
Pistachio	0.12	1 × 10^−4^	-	0.04	-	0.16
Walnut	7.2 × 10^−3^	3.8 × 10^−3^	-	0.12	-	0.13
Pulses-Beans						
Common bean white	0.12	1 × 10^−3^	-	0.08	8 × 10^−3^	0.2
Broad bean seed whole	-	8.9 × 10^−4^	-	0.09	-	0.09
Mung bean	-	-	-	0.18	-	0.18
Soy and soy products						
Soy paste, miso	0.02	3.6 × 10^−3^	-	0.01	-	0.03
Soy flour	-	7.5 × 10^−3^	-	0.3	-	0.3
Soy tempe	0.01	5 × 10^−4^	-	0.01	-	0.02
Soy tofu	0.04	7.27 × 10^−5^	8.5 × 10^−3^	9.91 × 10^−3^	0.04	0.09
Soy yogurt	0.01	3 × 10^−3^	-	0.02	-	0.03
Soyben edamame	0.07	-	0.02	0.07	0.2	0.3
Soybean sprout	0.03	5 × 10^−4^	0.01	0.03	0.05	0.12

**Table 3:** Lignan content of cereals (mg/100g food) [18].

	LAR	MAT	MED	SEC	SYR	Total
Cereal products						
Bread (whole grain flour)	0.05	3.1 × 10^−4^	-	8.68 × 10^−3^	-	0.05
Bread (refined flour)	0.01	1.23 × 10^−3^	-	7.19 × 10^−3^	0.04	0.05
Bread, rye, whole grain flour	0.01	0.02	-	0.14	-	0.17
Breakfast cereals, bran	0.01	4.87 × 10^−3^	-	0.03	-	0.04
Breakfast cereals, corn	-	1.67 × 10^−3^	-	5.5 × 10^−3^	-	0.007
Breakfast cereals, muesli	0.14	5.6 × 10^−3^	-	0.08	-	0.22
Breakfast cereal, oat	-	0.06	-	0.02	-	0.08
Pasta	-	1.85 × 10^−3^	-	2.3 × 10^−3^	-	0.004
Pasta Whole Grain	-	1.5 × 10^−3^	-	5 × 10^−3^	-	0.006
Cereals						
Barley, whole grain flour	0.08	3 × 10^−3^	0.01	0.03	0.16	0.28
Buckwheat, whole grain flour	0.36	1 × 10^−3^	0.03	0.13	0.24	0.76
Common wheat, germ	-	9 × 10^−3^	-	0.02	-	0.02
Common wheat, refined flour	0.18	2.14 × 10^−4^	-	0.02	-	0.2
Common wheat, whole grain flour	0.1	9 × 10^−4^	0.03	0.02	0.37	0.52
Hard wheat, semolin	-	-	-	2 × 10^−3^	-	0.002
Maize, whole grain	0.12	6.55 × 10^−5^	-	0.14	0.07	0.33
Oat, whole grain flour	0.18	0.07	0.04	0.01	0.35	0.65
Rye, whole grain flour	0.32	0.01	0.14	0.02	0.97	1.46

**Table 4:** Lignan contents of vegetables (mg/100g food) [18].

	LAR	MAT	MED	SEC	SYR	Total
Cabbages						
Broccoli	97.2	2.44 × 10^−5^	-	1.31	-	98.51
Brussel sprouts	49.3	4 × 10^−5^	-	1.06	-	50.36
Cauliflower	9.31	2.4 × 10^−5^	0.02	0.13	0.02	9.48
Collards	0.06	4 × 10^−4^	-	5.9 × 10^−3^	-	0.06
Green cabbage	0.03	3.5 × 10^−5^	-	9.2 × 10^−3^	-	0.03
Red cabbage	17.8	4.44 × 10^−5^	-	0.3	-	18.1
White cabbage	21.2	-	-	0.31	-	21.51
Kale	59.9	1.2	-	1.9	-	63
Sauerkraut	11.6	-	-	6.7	-	18.3
Fruit vegetales						
Avocado	0.03	7.67 × 10^−3^	0.24	0.02	0.44	0.73
Eggplant purple	0.05	-	7 × 10^−3^	7.79 × 10^−3^	6 × 10^−3^	0.07
Black olive	0.03	5.62 × 10^−3^	-	5.75 × 10^−3^	-	0.04
Green olive	3.9 × 10^−3^	3.34 × 10^−3^	-	0.02	-	0.02
Green sweet pepper	12.32	-	1 × 10^−3^	0.22	4 × 10^−3^	12.54
Red sweet pepper	7.97	-	-	0.24	-	8.21
Yellow sweet pepper	0.07	-	-	5.5 × 10^−3^	-	0.07
Tomato (Cherry)	0.03	-	3 × 10^−3^	0.01	4.5 × 10^−3^	0.04
Tomato (Whole)	2.1	8.33 × 10^−6^	3.5 × 10^−3^	0.05	4.5 × 10^−3^	2.15
Gourds						
Cucumber	3.55	-	-	0.25	-	3.8
Pumpkin	0.01	2.5 × 10^−5^	-	0.1	-	0.11
Squash	-	-	-	9 × 10^−3^	-	0.009
Zucchini	6.4	-	-	0.62	-	7.02
Leaf vegetables						
Arugula	-	2 × 10^−4^	-	0.1	-	0.1
Chicory (green)	0.6	1.24 × 10^−4^	-	0.57	-	1.17
Lettuce (green)	0.3	2.24 × 10^−4^	-	0.18	-	0.48
Spinach	0.06	2.37 × 10^−5^	-	4.85 × 10^−3^	-	0.06
Broad bean pod	-	-	-	0.02	-	0.02
Pod vegetables						
Green bean	22	-	-	0.67	-	22.67
Pulse vegetables						
Fresh pea	0.05	-	3.5 × 10^−3^	7.56 × 10^−4^	-	0.0542
Root vegetables						
Carrot	4.5	3.89 × 10^−3^	-	3.16	-	7.66
Celeriac	-	3 × 10^−5^	-	0.02	-	0.02
Parsnip	-	0.02	-	0.03	-	0.05
Radish	0.01	1.25 × 10^−4^	5.5 × 10^−3^	6.57 × 10^−3^	0.02	0.04
Swede	-	7.43 × 10^−5^	-	4.93 × 10^−3^	-	0.005
Turnip root	0.1	-	4 × 10^−3^	9.83 × 10^−3^	0.03	0.14
Shoot vegetables						
Asparagus	0.07	3.97 × 10^−3^	4 × 10^−3^	0.25	0.05	0.37
Fennel	-	0.01	-	0.05	-	0.06
Stalks vegetables						
Celery stalks	-	-	-	5.99 × 10^−3^	-	0.005
Tubers						
Potato	2.8	7.69 × 10^−4^	-	0.09	-	2.89
Sweet potato	0.07	0.1	-	0.12	-	0.29

**Table 5:** Lignan contents of fruits berries (mg/100g food) [18].

	**HMA**	**OXO**	**CON**	**CYC**	**LAR**	**LAS**
Fruit Berries						
Bilberry	-	-	-	6.24 × 10^−3^	0.04	0.09
Blackberry	-	-	-	7.96 × 10^−3^	0.15	0.15
Blackcurrant	-	-	-	0.01	7.3 × 10^−3^	0.01
Cloudberry	-	-	-	-	0.65	0.25
Black grape	-	-	-	-	5.2	-
Green grape	-	-	-	-	1.88	-
Lingonberry	-	-	1.04 × 10^−3^	0.03	0.03	0.01
Strawberry	8.55 × 10^−4^	4.59 × 10^−4^	9.45 × 10^−3^	0.01	5.87	0.1
	**MAT**	**MED**	**SEC**	**SECS**	**SYR**	**Total**
Bilberry	-	0.08	0.06	0.01	0.12	0.4
Blackberry	9.07 × 10^−4^	0.05	0.1	0.13	0.19	0.77
Blackcurrant	1.47 × 10^−3^	0.01	0.09	0.03	-	0.15
Cloudberry	-	0.48	0.05	0.01	0.41	1.85
Black grape	0.11	-	0.09	-	-	5.4
Green grape	0.09	-	0.28	-	-	2.25
Lingonberry	-	0.23	0.37	0.02	0.14	0.83
Strawberry	1.58 × 10^−5^	0.03	0.14	0.01	0.03	6.2

**Table 6:** Lignan contents of fruits (mg/100g food) [18].

	**LAR**	**MAT**	**MED**	**SEC**	**SYR**	**Total**
Fruits Citrus						
Grapefruit	7.13	0.05	-	0.26	-	7.44
Lemon	-	-	-	0.02	-	0.02
Orange	2.4	0.05	9.5 × 10^−3^	0.14	0.12	2.71
Tangerine	5.7	0.02	-	0.08	-	5.8
Fruits Drupes						
Apricot	10.5	3.11 × 10^−5^	-	1.07	-	11.57
Nectarine	4.1	-	-	0.61	-	4.71
Peach	6	1.71 × 10^−4^	-	0.83	-	6.83
Plum	0.31	2.22 × 10^−4^	1 × 10^−3^	0.09	-	0.4
Fruits-Gourds						
Cantaloupe	1.8 × 10^−3^	-	-	4.7 × 10^−3^	-	0.006
Melon	4.4	1.05 × 10^−5^	-	0.09	-	4.49
Watermelon	0.04	-	1 × 10^−3^	0.02	0.02	0.08
Fruits-Pomes						
Apple	0.1	2.71 × 10^−5^	-	1.79 × 10^−3^	-	0.1
Pear	15.5	4.3 × 10^−5^	-	0.06	-	15.56
Fruits-Tropical						
Banana	2.2 × 10^−3^	5.45 × 10^−5^	-	7.73 × 10^−5^	0.01	0.01
Kiwi	1.03	1.93 × 10^−3^	4.5 × 10^−3^	3.13	4 × 10^−3^	4.17
Mango	-	1.06 × 10^−3^	-	0.01	-	0.01
Passion fruit	-	-	-	0.02	-	0.02
Papaya	-	2 × 10^−3^	-	-	-	0.002
Persimmon	-	-	-	4 × 10^−3^	-	0.004
Pineapple	0.2	0.16	2 × 10^−3^	0.21	0.09	0.66
Pomegranate	-	9 × 10^−3^	-	0.29	-	0.29

**Table 7:** Lignan content of beverages (mg/100g drink and mg/100 mL wine) [18].

	**ISO**	**LAR**	**MAT**	**SEC**	**SYR**	**Total**
Alcoholic Beverages						
Red Wine	0.07	7.56 × 10^−3^	5.51 × 10^−3^	0.04	3.43 × 10^−3^	0.12
White Wine	0.03	6.65 × 10^−3^	2.68 × 10^−3^	7.45 × 10^−3^	1.45 × 10^−3^	0.04
Dark Beer	-	-	-	0.04	-	0.04
Beer	-	-	-	0.03	-	0.03
Cider	-	-	-	0.04	-	0.04
Scotch whisky	-	-	-	4 × 10^−3^	-	0.004
Sherry	-	-	-	0.02	-	0.02
Non-alcoholic Beverages						
Cocoa	-	-	-	0.03	-	0.03
Coffee	-	9 × 10^−4^	4 × 10^−4^	8.67 × 10^−3^	-	0.009
Decaffeinated Coffe	-	1.1 × 10^−3^	4.25 × 10^−4^	8.35 × 10^−3^	-	0.009
Roman camomile	-	-	5 × 10^−4^	1 × 10^−3^	-	0.001
Lemon juice	-	-	-	2 × 10^−3^	-	0.002
Orange juice	-	2 × 10^−4^	-	8 × 10^−3^	-	0.008
Soy milk	-	6.17 × 10^−3^	5 × 10^−5^	2.25 × 10^−3^	-	0.008
Black Tea	-	2 × 10^−4^	2.65 × 10^−3^	0.03	-	0.03
Green Tea	-	1 × 10^−4^	3.38 × 10^−3^	0.03	-	0.03
Oolong Tea	-	-	1.8 × 10^−3^	0.02	-	0.02

**Table 8:** Lignan content of oils (mg/100 g food) [18].

**Fruit oils**	**ACE**	**LAR**		**MAT**		**PIN**	**SEC**	**Total**
Extra virgin Olive Oil	0.66	3.43 × 10^−3^		7.5 × 10^−5^		0.42	2.5 × 10^−4^	1.08
Nut oils								
Peanut, butter	-	8.8 × 10^−3^		7.52 × 10^−3^		-	0.05	0.06
**Other seed oils**	**EPI**	**EPL**	**SES**	**SEI**	**SEO**	**SEN**	**SEL**	**Total**
Sesame seed oil	192.6	51.97	420.99	305.43	24.92	243.13	55.71	1294.75
Sesame seed black oil	-	-	644.5	226.92	21.55	287.33	43	1223.3

1-Acetoxypinoresinol (ACE), Episesamin (EPI), Episesaminol (EPL), Pinoresinol (PIN), Sesamol (SEO), Sesamolinol (SEL).

**Table 9:** Association between naturally lignan-rich foods and health promotion.

Author, Year	Methods	Results
Breast Cancer		
Lowcock, E.C. et al. (2013) [111]	Case-control study (2999 cases and 3370 controls) FFQ	Consumption of flaxseed and flax bread was associated with a significant reduction in breast cancer risk (OR 0.82, 95% CI 0.69–0.97; and OR 0.77, 95% CI 0.67–0.89), respectively.
McCann et al. (2012) [113]	Case-control study (638 cases and 611 controls) BioRepository at Roswell Park Cancer Institute FFQ	Lignan intakes were inversely associated with risk of ER (−) breast cancer among premenopausal women (OR 0.16, 95% CI 0.03–0.44) and particularly triple negative tumors (OR 0.16, 95% CI 0.04–0.62).
Zaineddin AK et al. (2012) [114]	Case-control study (2884 cases and 5509 controls) FFQ	High and low consumption of soybeans, as well as of sunflower and pumpkin seeds were associated with significantly reduced breast cancer risk compared to no consumption (OR 0.83, 95% CI 0.70–0.97; and OR 0.66, 95% CI 0.77–0.97, respectively).
Buck K et al. (2011) [112]	1140 postmenopausal patients (age 50 to 74 years) FFQ Serum Enterolactone	Serum enterolactone was associated with a significantly reduced risk of death only for estrogen receptor-negative tumors (HR 0.27; 95% CI 0.08 to 0.87)
Buck K et al. (2010) [116]	Meta-analyses Medline search to identify epidemiologic studies published between 1997 and August 2009	Lignan exposure was not associated with overall breast cancer risk (RE 0.92; 95% CI 0.81, 1.02).
McCann, S.E et al. (2010) [107]	Breast cancer patients; National Death Index Food frequency questionnaire (FFQ), DietSys (3.7)	Lignan intake among post-menopausal women with breast cancer significantly reduced risk of mortality from breast cancer (HR 0.29, 95% CI, 0.11–0.76), as well as significantly reducing risk of all-cause mortality (HR 0.49, 95% CI 0.26–0.91).
Velentzis LS et al. (2009) [115]	Meta-analy sesMedline, BIOSIS and EMBASE databases publications up to 30 September 2008	Overall, there was little association between high plant lignan intake and breast cancer risk (11 studies, OR 0.93, 95% CI 0.83–1.03).
Cotterchio, M et al. (2008) [109]	Ontario Cancer Registry; Controls: Age-stratified random sample of women FFQ	Total phytoestrogen intake in pre-menopausal women was associated with a significant reduction in breast cancer risk among overweight women (OR 0.51, 95% CI 0.30, 0.87).
Suzuki, R. et al. (2008) [108]	Swedish Mammography Cohort FFQ and Swedish National Food database Serum Enterolactone: Fluoroimmunoassay Receptor status of tumors: Immunohistochemical	A significant 17% risk reduction for breast cancer overall in high lignan intake was observed, but no heterogeneity across Estrogen Receptor/Progesterone Receptor subtypes.
Trock BJ et al. (2006) [110]	Meta-analysis of 18 epidemiologic studies published from 1978 through 2004	High soy intake was discreetly associated with reduction of breast cancer risk (OR 0.86, 95% CI: 0.75 to 0.99); association was not statistically significant among women in Asian countries (OR 0.89, 95% CI 0.71 to 1.12).
Gastroesophageal Cancer		
Lin Y et al. (2012) [117]	Case-control study (1995–1997); 806 controls, 181 cases of esophageal adenocarcinoma, 255 cases of gastroesophageal junctional adenocarcinoma, and 158 cases of esophageal squamous cell carcinoma. Interviews and questionnaires; FFQ	No clear associations were found between risk of esophageal carcinoma and lignan intake.
Lin Y et al. (2012) [118]	Cohort study in Sweden, 81,670 (followed up 1998 to 2009). Cancer cases: Swedish Cancer Register FFQ	There was no statistically significant association between dietary intake of lignans and any of the studied adenocarcinomas.
Colon Cancer		
Zamora-Ros, R. et al. (2015) [119]	409 CRC cases in Barcelona (Spain). FFQ; Phenol-Explorer database.	No associations were also observed with either total lignans or any flavonoid subclass intake.
Prostate Cancer		
Wallström P et al. (2018) [120]	Case-control study (1010 cases and 1817 controls) National registers and hospital records FFQ Plasma Enterolactone: Fluoroimmunoassay	There were no significant associations between plasma enterolactone and incidence of prostate cancer (OR 0.99, 95% CI 0.77–1.280)
Eriksen AK et al. (2017) [121]	1390 men diagnosed with prostate cancer from the Danish Diet, Cancer and Health cohort Plasma Enterolactone: Fluoroimmunoassay	No associations between plasma enterolactone concentrations and prostate cancer aggressiveness.
Hedelin M et al. (2006) [123]	Swedish case-control study (1499 prostate cancer cases and 1130 controls) FFQ	No association was found between dietary intake of total or individual lignans or isoflavonoids and risk of prostate cancer.
Bylund A. et al. (2003) [122]	10 men with prostate cancer were randomized to a daily supplement of rye bran bread and 8 men of wheat bread Blood and urine samples. Ultrasound-guided core biopsies of the prostate.	In the rye group, there was a significant increase in plasma enterolactone. However, only small changes were observed in plasma concentrations of prostate specific antigen (PSA).
Cardiovascular disease		
Witkowska AM et al. (2018) [126]	2599 postmenopausal women, participants of the Multi-center National Population Health Examination Surveys. 24-h Dietary recall and food databases.	In postmenopausal women, total and individual lignan intakes (secoisolariciresinol, pinoresinol, matairesinol) were not associated with the prevalence of CVD and its risk factors.
Pellegrini N et al. (2010) [127]	Cross-sectional study in 151 men and 91 post-menopausal women. Anthropometric characteristics. Soluble intercellular adhesion molecule-1 (sICAM-1), CRP, insulin, glucose, total cholesterol, HDL-cholesterol and triacylglycerols. Three-day weighed food record	No relationship between intake of pinoresinol, lariciresinol or total lignans and sICAM-1 values was observed.
Jacobs DR. et al. (2000) [128]	11,040 postmenopausal women enrolled in the Iowa Women’s Health Study Followed from baseline 1986−997.	Women who consumed on average 1.9 g refined grain fiber/2000 kcal and 4.7 g whole grain fiber/2000 kcal had a 17% lower mortality rate (RR = 0.83, 95% CI = 0.73–0.94) than women who consumed predominantly refined grain fiber.
Vanharanta M. et al. (2003) [129]	A prospective study of Finnish men. 1889 men aged 42 to 60 years. Followed up 12.2 years.	Multivariate analyses showed significant associations between elevated serum enterolactone concentration and reduced risk of CVD-related mortality.
Other diseases		
Franco OH. et al. (2005) [130]	Community-based survey among 394 postmenopausal women. FFQ; Cognitive function:Mini-Mental Examination	Increasing dietary lignans intake was associated with better performance on the MMSE (OR 1.49, 95% CI 0.94–2.38). Results were most pronounced in women who were 20–30 years.
Eichholzer M. et al. (2014) [131]	2028 participants of NHANES 2005-2008 and 2628 participants of NHANES 1999-2004 (aged ≥18 years) Inflammatory marker: CRP	Statistically significant inverse associations of urinary lignan, enterodiol, and enterolactone concentrations with circulating CRP counts were observed in the multivariate-adjusted models.

FFQ: Food Frequency Questionnaire; CI: Confidence Interval; HR: Hazard Ratio; OR: Odds Ratio; CVD: Cardiovascular Disease; MMSE: Cognitive function Mini-Mental Examination; CRP: C-Reactive Protein.

## References

[1] Marilena V., Olga V., Maria M., Enzo B., Stefano D.P., Carlo B., Giorda A.N., Sebastiano S., Stefania A., Anna C. (2017). Rivellese, Polyphenol intake, cardiovascular risk factors in a population with type 2 diabetes: The TOSCA.IT study. Clin. Nutr..

[2] Rocha L., Monteiro M., Anderson T. (2012). Anticancer Properties of Hydroxycinnamic Acids-A Review. Cancer Clin. Oncol..

[3] Adlercreutz H. (2007). Lignans, human health. Crit. Rev. Clin. Lab. Sci..

[4] Ionkova I. (2011). Anticancer lignans—From discovery to biotechnology. Mini Rev. Med. Chem..

[5] Peterson J., Dwyer J., Adlercreutz H., Scalbert A., Jacques P., McCullough M.L. (2010). Dietary lignans: Physiology, potential for cardiovascular disease risk reduction. Nutr. Rev..

[6] Landete J.M. (2012). Plant, mammalian lignans: A review of source, intake, metabolism, intestinal bacteria, health. Food Res. Int..

[7] Touré A., Xu X. (2010). Flaxseed Lignans: Source, Biosynthesis, Metabolism, Antioxidant Activity, Bio-Active Components, Health Benefits. Compr. Rev. Food Sci. Food Saf..

[8] Marcotullio M.C., Curini M., Becerra J.X. (2018). An Ethnopharmacological, Phytochemical, Pharmacological Review on Lignans from *Mexican Bursera* spp.. Molecules.

[9] Magoulas G.E., Papaioannou D. (2014). Bioinspired syntheses of dimeric hydroxycinnamic acids (lignans), hybrids, using phenol oxidative coupling as key reaction, medicinal significance thereof. Molecules.

[10] Li Y., Xie S., Ying J., Wei W., Gao K. (2018). Chemical Structures of Lignans, Neolignans Isolated from Lauraceae. Molecules.

[11] Pan J.Y., Chen S.L., Yang M.H., Wu J., Sinkkonen J., Zou K. (2009). An update on lignans: Natural products, synthesis. Nat. Prod. Rep..

[12] Suzuki S., Umezawa T. (2007). Biosynthesis of lignans, norlignans. J. Wood Sci..

[13] Solyomvary A., Beni S., Boldizsar I. (2017). Dibenzylbutyrolactone Lignans-A Review of Their Structural Diversity, Biosynthesis, Occurrence, Identification, Importance. Mini Rev. Med. Chem..

[14] Xu W.-H., Zhao P., Wang M., Liang Q. (2018). Naturally occurring furofuran lignans: Structural diversity, biological activities. Nat. Prod. Res..

[15] Zhang J., Chen J., Liang Z., Zhao C. (2014). New lignans, their biological activities. Chem. Biodivers.

[16] Durazzo A., Zaccaria M., Polito A., Maiani G., Carcea M. (2013). Lignan Content in Cereals, Buckwheat, Derived Foods. Foods.

[17] Durazzo A., Turfani V., Azzini E., Maiani G., Carcea M. (2013). Phenols, lignans, antioxidant properties of legume, sweet chestnutflours. Food Chem..

[18] Rothwell J.A., Pérez-Jiménez J., Neveu V., Medina-Ramon A., M’Hiri N., Garcia Lobato P., Manach C., Knox K., Eisner R., Wishart D. (2013). Phenol-Explorer 3.0: A major update of the Phenol-Explorer database to incorporate data on the effects of food processing on polyphenol content. Database.

[19] Smeds A.I., Jauhiainen L., Tuomola E. (2009). Peltonen-Sainio, P. Characterization of variation in the lignan content, composition of winter rye, spring wheat, spring oat. J. Agric. Food Chem..

[20] Esposito F., Arlotti G., Maria Bonifati A., Napolitano A., Vitale D., Fogliano V. (2005). Antioxidant activity, dietary fibre in durum wheat bran by-products. Food Res. Int..

[21] Fardet A. (2010). New hypotheses for the health-protective mechanisms of whole-grain cereals: What is beyond fibre?. Nutr. Res. Rev..

[22] Bolvig A.K., Adlercreutz H., Theil P.K., Jorgensen H., Bach Knudsen K.E. (2016). Absorption of plant lignans from cereals in an experimental pig model. Br. J. Nutr..

[23] Milder I.E., Arts I.C., van de Putte B., Venema D.P., Hollman P.C. (2005). Lignan contents of Dutch plant foods: A database including lariciresinol, pinoresinol, secoisolariciresinol, matairesinol. Br. J. Nutr..

[24] Ruiz-Aracama A., Goicoechea E., Guillén M.D. (2017). Direct study of minor extra-virgin olive oil components without any sample modification. 1H NMR multisupression experiment: A powerful tool. Food Chem..

[25] Ricciutelli M., Marconi S., Boarelli M.C., Caprioli G., Sagratini G., Ballini D., Fiorini R. (2017). Olive oil polyphenols: A quantitative method by high-performance liquid-chromatography-diode-array detection for their determination, the assessment of the related health claim. J. Chromatogr. A.

[26] Milder I.E., Feskens E.J., Arts I.C., Bueno de Mesquita H.B., Hollman P.C., Kromhout D. (2005). Intake of the plant lignans secoisolariciresinol, matairesinol, lariciresinol, pinoresinol in Dutch men, women. J. Nutr..

[27] Sun Q., Wedick N.M., Pan A., Townsend M.K., Cassidy A., Franke A.A., Rimm E.B., Hu F.B., van Dam R.M. (2014). Gut microbiota metabolites of dietary lignans, risk of type 2 diabetes: A prospective investigation in two cohorts of U.S. women. Diabetes Care.

[28] McCann M.J., Gill C.I., McGlynn H., Rowland I.R. (2005). Role of mammalian lignans in the prevention, treatment of prostate cancer. Nutr. Cancer.

[29] Szewczyk M., Abarzua S., Schlichting A.E., Nebe B., Piechulla B., Volker B., Dagmar-Ulrike R. (2014). Effects of extracts from Linum usitatissimum on cell vitality, proliferation, cytotoxicity in human breast cancer cell lines. J. Med. Plant Res..

[30] Björck I., Östman E., Kristensen M., Mateo Anson N., Price R.K., Haenen G.R.M.M., Havenaar R., Bach Knudsen K.E., Frid A., Mykkänen H. (2012). Cereal grains for nutrition, health benefits: Overview of results from in vitro, animal, human studies in the HEALTHGRAIN project. Trends Food Sci. Technol..

[31] Kuijsten A., Arts I.C., Vree T.B., Hollman P.C. (2005). Pharmacokinetics of enterolignans in healthy men, women consuming a single dose of secoisolariciresinol diglucoside. J. Nutr..

[32] Tetens I., Turrini A., Tapanainen H., Christensen T., Lampe J.W., Fagt S., Håkansson N., Lundquist A., Hallund J., Valsta L.M. (2013). Dietary intake, main sources of plant lignans in five European countries. Food Nutr. Res..

[33] Heinonen S., Nurmi T., Liukkonen K., Poutanen K., Wahala K., Deyama T., Nishibe S., Adlercreutz H. (2001). In vitro metabolism of plant lignans: New precursors of mammalian lignans enterolactone, enterodiol. J. Agric. Food Chem..

[34] Saarinen N.M., Thompson L.U. (2010). Prolonged administration of secoisolariciresinol diglycoside increases lignan excretion, alters lignan tissue distribution in adult male, female rats. Br. J. Nutr..

[35] Mukker J.K., Singh R.S., Muir A.D., Krol E.S., Alcorn J. (2015). Comparative pharmacokinetics of purified flaxseed, associated mammalian lignans in male Wistar rats. Br. J. Nutr..

[36] Chaojie L., Ed S.K., Jane A. (2013). The Comparison of Rat, Human Intestinal, Hepatic Glucuronidation of Enterolactone Derived from Flaxseed Lignans. Nat. Prod. J..

[37] Murray T., Kang J., Astheimer L., Price W.E. (2007). Tissue distribution of lignans in rats in response to diet, dose-response, competition with isoflavones. J. Agric. Food Chem..

[38] Thompson L.U., Chen J.M., Li T., Strasser-Weippl K., Goss P.E. (2005). Dietary flaxseed alters tumor biological markers in postmenopausal breast cancer. Clin. Cancer Res..

[39] Clavel T., Dore J., Blaut M. (2006). Bioavailability of lignans in human subjects. Nutr. Res. Rev..

[40] Adlercreutz H. (2002). Phyto-oestrogens, cancer. Lancet. Oncol..

[41] Kuijsten A., Arts I.C., van’t Veer P., Hollman P.C. (2005). The relative bioavailability of enterolignans in humans is enhanced by milling, crushing of flaxseed. J. Nutr..

[42] Lærke H.N., Mortensen M.A., Hedemann M.S., Bach Knudsen K.E., Penalvo J.L., Adlercreutz H. (2009). Quantitative aspects of the metabolism of lignans in pigs fed fibre-enriched rye, wheat bread. Br. J. Nutr..

[43] Johnson T.W., Dress K.R., Edwards M. (2009). Using the Golden Triangle to optimize clearance, oral absorption. Bioorganic. Med. Chem. Lett..

[44] Li J.J., Cheng L., Shen G., Qiu L., Shen C.Y., Zheng J., Xu R., Yuan H.L. (2018). Improved stability, oral bioavailability of Ganneng dropping pills following transforming lignans of herpetospermum caudigerum into nanosuspensions. Chin. J. Nat. Med..

[45] Tierney A.C., Zabetakis I. (2018). Changing the Irish dietary guidelines to incorporate the principles of the Mediterranean diet: Proposing the MedEire diet. Public Health Nutr..

[46] Trichopoulou A., Costacou T., Bamia C., Trichopoulos D. (2003). Adherence to a Mediterranean Diet, Survival in a Greek Population. N Engl. J. Med..

[47] Pounis G., Di Castelnuovo A., Bonaccio M., Costanzo S., Persichillo M., Krogh V., Donati M.B., de Gaetano G., Iacoviello L. (2016). Flavonoid, lignan intake in a Mediterranean population: Proposal for a holistic approach in polyphenol dietary analysis, the Moli-sani Study. Eur. J. Clin. Nutr..

[48] Bolvig A.K., Norskov N.P., van Vliet S., Foldager L., Curtasu M.V., Hedemann M.S., Sorensen J.F., Laerke H.N., Bach Knudsen K.E. (2017). Rye Bran Modified with Cell Wall-Degrading Enzymes Influences the Kinetics of Plant Lignans but Not of Enterolignans in Multicatheterized Pigs. J. Nutr..

[49] Bolling B.W., Chen C.Y., McKay D.L., Blumberg J.B. (2011). Tree nut phytochemicals: Composition, antioxidant capacity, bioactivity, impact factors. A systematic review of almonds, Brazils, cashews, hazelnuts, macadamias, pecans, pine nuts, pistachios, walnuts. Nutr. Res. Rev..

[50] Guasch-Ferré M., Hu F.B., Martínez-González M.A., Fitó M., Bulló M., Estruch R., Ros E., Corella D., Recondo J., Gómez-Gracia E. (2014). Olive oil intake, risk of cardiovascular disease, mortality in the PREDIMED Study. BMC Med..

[51] Toledo E., Salas-Salvado J., Donat-Vargas C., Buil-Cosiales P., Estruch R., Ros E., Corella D., Fito M., Hu F.B., Aros F.E. (2015). Mediterranean Diet, Invasive Breast Cancer Risk Among Women at High Cardiovascular Risk in the PREDIMED Trial: A Randomized Clinical Trial. JAMA Int. Med..

[52] Medina-Remón A., Casas R., Tressserra-Rimbau A., Ros E., Martínez-González M.A., Fitó M., Corella D., Salas-Salvadó J., Lamuela-Raventos R.M., Estruch R. (2017). Polyphenol intake from a Mediterranean diet decreases inflammatory biomarkers related to atherosclerosis: A substudy of the PREDIMED trial. Br. J. Clin. Pharmacol..

[53] Lopez-Biedma A., Sanchez-Quesada C., Beltran G., Delgado-Rodriguez M., Gaforio J.J. (2016). Phytoestrogen (+)-pinoresinol exerts antitumor activity in breast cancer cells with different oestrogen receptor statuses. BMC Compl. Altern. Med..

[54] Antonini E., Farina A., Scarpa E.S., Frati A., Ninfali P. (2016). Quantity, quality of secoiridoids, lignans in extra virgin olive oils: The effect of two-, three-way decanters on Leccino, Raggiola olive cultivars. Int. J. Food Sci. Nutr..

[55] Ramezani-Jolfaie N., Mohammadi M., Salehi-Abargouei A. (2018). The effect of healthy Nordic diet on cardio-metabolic markers: A systematic review, meta-analysis of randomized controlled clinical trials. Eur. J. Nutr..

[56] Galbete C., Kröger J., Jannasch F., Iqbal K., Schwingshackl L., Schwedhelm C., Weikert C., Boeing H., Schulze M.B. (2018). Nordic diet, Mediterranean diet, the risk of chronic diseases: The EPIC-Potsdam study. BMC Med..

[57] Smeds A.I., Eklund P.C., Sjoholm R.E., Willfor S.M., Nishibe S., Deyama T., Holmbom B.R. (2007). Quantification of a broad spectrum of lignans in cereals, oilseeds, nuts. J. Agric. Food Chem..

[58] Konye R., Toth G., Solyomvary A., Mervai Z., Zurn M., Baghy K., Kovalszky I., Horvath P., Molnar-Perl I., Noszal B. (2018). Chemodiversity of Cirsium fruits: Antiproliferative lignans, neolignans, sesquineolignans as chemotaxonomic markers. Fitoterapia.

[59] Boldizsar I., Kraszni M., Toth F., Noszal B., Molnar-Perl I. (2010). Complementary fragmentation pattern analysis by gas chromatography-mass spectrometry, liquid chromatography tandem mass spectrometry confirmed the precious lignan content of Cirsium weeds. J. Chromatogr. A.

[60] Singh L., Agarwal T. (2018). PAHs in Indian diet: Assessing the cancer risk. Chemosphere.

[61] Liu W.J., Chen Y., Chen D., Wu Y., Gao Y.J., Li J., Zhong W.J., Jiang L. (2018). A new pair of enantiomeric lignans from the fruits of Morinda citrifolia, their absolute configuration. Nat. Prod. Res..

[62] Nguyen P.H., Yang J.L., Uddin M.N., Park S.L., Lim S.I., Jung D.W., Williams D.R., Oh W.K. (2013). Protein tyrosine phosphatase 1B (PTP1B) inhibitors from Morinda citrifolia (Noni), their insulin mimetic activity. J. Nat. Prod..

[63] Chen J., Chen Y., Tian J., Ge H., Liang X., Xiao J., Lin H. (2018). Simultaneous determination of four sesame lignans, conversion in Monascus aged vinegar using HPLC method. Food Chem..

[64] Yashaswini P.S., Sadashivaiah B., Ramaprasad T.R., Singh S.A. (2017). In vivo modulation of LPS induced leukotrienes generation, oxidative stress by sesame lignans. J. Nutr. Biochem..

[65] Namiki M. (2007). Nutraceutical functions of sesame: A review. Crit. Rev. Food Sci. Nutr..

[66] Dar A.A., Arumugam N. (2013). Lignans of sesame: Purification methods, biological activities, biosynthesis—A review. Bioorganic Chem..

[67] Katayama S., Sugiyama H., Kushimoto S., Uchiyama Y., Hirano M., Nakamura S. (2016). Effects of Sesaminol Feeding on Brain Aβ Accumulation in a Senescence-Accelerated Mouse-Prone 8. J. Agric. Food Chem..

[68] Liu Z., Saarinen N.M., Thompson L.U. (2006). Sesamin Is One of the Major Precursors of Mammalian Lignans in Sesame Seed (Sesamum indicum) as Observed In Vitro, in Rats. J. Nutr..

[69] Hsu W.C., Lau K.H.K., Matsumoto M., Moghazy D., Keenan H., King G.L. (2014). Improvement of Insulin Sensitivity by Isoenergy High Carbohydrate Traditional Asian Diet: A Randomized Controlled Pilot Feasibility Study. PLoS ONE.

[70] Su S., Wink M. (2015). Natural lignans from Arctium lappa as antiaging agents in Caenorhabditis elegans. Phytochemistry.

[71] Kou X., Qi S., Dai W., Luo L., Yin Z. (2011). Arctigenin inhibits lipopolysaccharide-induced iNOS expression in RAW264.7 cells through suppressing JAK-STAT signal pathway. Int. Immunopharmacol..

[72] Susanti S., Iwasaki H., Inafuku M., Taira N., Oku H. (2013). Mechanism of arctigenin-mediated specific cytotoxicity against human lung adenocarcinoma cell lines. Phytomedicine.

[73] Zhang Y., Wang K., Chen H., He R., Cai R., Li J., Zhou D., Liu W., Huang X., Yang R. (2018). Anti-inflammatory lignans, phenylethanoid glycosides from the root of Isodon ternifolius (D.Don) Kudô. Phytochemistry.

[74] Chen Y., Tang Y.M., Yu S.L., Han Y.W., Kou J.P., Liu B.L., Yu B.Y. (2015). Advances in the pharmacological activities, mechanisms of diosgenin. Chin. J. Nat. Med..

[75] Chen F., Li C., Ma J., Ni L., Huang J., Li L., Lin M., Hou Q., Zhang D. (2018). Diterpenoids, lignans from the leaves of Tripterygium wilfordii. Fitoterapia.

[76] Xu J., Lu J., Sun F., Zhu H., Wang L., Zhang X., Ma Z. (2011). Terpenoids from Tripterygium wilfordii. Phytochemistry.

[77] Hu D., Yang Z., Yao X., Wang H., Han N., Liu Z., Wang Y., Yang J., Yin J. (2014). Dibenzocyclooctadiene lignans from Schisandra chinensis, their inhibitory activity on NO production in lipopolysaccharide-activated microglia cells. Phytochemistry.

[78] Yang B.Y., Han W., Han H., Liu Y., Guan W., Li X.M., Kuang H.X. (2018). Effects of Lignans from Schisandra chinensis Rattan Stems against Abeta1-42-Induced Memory Impairment in Rats, Neurotoxicity in Primary Neuronal Cells. Molecules.

[79] Wang O., Cheng Q., Liu J., Wang Y., Zhao L., Zhou F., Ji B. (2014). Hepatoprotective effect of Schisandra chinensis (Turcz.) Baill. lignans, its formula with Rubus idaeus on chronic alcohol-induced liver injury in mice. Food Funct..

[80] Panossian A., Wikman G. (2008). Pharmacology of Schisandra chinensis Bail.: An overview of Russian research, uses in medicine. J. Ethnopharmacol..

[81] Wu W., Ruan H. (2018). Triterpenoids, lignans from the stems of Schisandra glaucescens. Nat. Prod. Res..

[82] Yu H.Y., Chen Z.Y., Sun B., Liu J., Meng F.Y., Liu Y., Tian T., Jin A., Ruan H.L. (2014). Lignans from the fruit of Schisandra glaucescens with antioxidant, neuroprotective properties. J. Nat. Prod..

[83] Liu P., Kallio H., Yang B. (2011). Phenolic Compounds in Hawthorn (*Crataegus grayana*) Fruits, Leaves, Changes during Fruit Ripening. J. Agric. Food Chem..

[84] Huang X.-X., Bai M., Zhou L., Lou L.-L., Liu Q.-B., Zhang Y., Li L.-Z., Song S.-J. (2015). Food Byproducts as a New, Cheap Source of Bioactive Compounds: Lignans with Antioxidant, Anti-inflammatory Properties from Crataegus pinnatifida Seeds. J. Agric. Food Chem..

[85] Jiang K., Song Q.Y., Peng S.J., Zhao Q.Q., Li G.D., Li Y., Gao K. (2015). New lignans from the roots of Schisandra sphenanthera. Fitoterapia.

[86] Liu Y., Yang Y., Tasneem S., Hussain N., Daniyal M. (2018). Lignans from Tujia Ethnomedicine Heilaohu: Chemical Characterization, Evaluation of Their Cytotoxicity, Antioxidant Activities. Molecules.

[87] Sun J., Yao J., Huang S., Long X., Wang J., García-García E. (2009). Antioxidant activity of polyphenol, anthocyanin extracts from fruits of *Kadsura coccinea* (Lem.) A.C. Smith. Food Chem..

[88] Kim K.H., Choi J.W., Ha S.K., Kim S.Y., Lee K.R. (2010). Neolignans from Piper kadsura, their anti-neuroinflammatory activity. Bioorg. Med. Chem. Lett..

[89] Li W., Sun Y.N., Yan X.T., Yang S.Y., Kim E.J., Kang H.K., Kim Y.H. (2013). Coumarins, lignans from Zanthoxylum schinifolium, their anticancer activities. J. Agric. Food Chem..

[90] Cui H.Z., Choi H.R., Choi D.H., Cho K.W., Kang D.G., Lee H.S. (2009). Aqueous extract of Zanthoxylum schinifolium elicits contractile, secretory responses via beta1-adrenoceptor activation in beating rabbit atria. J. Ethnopharmacol..

[91] Min B.K., Hyun D.G., Jeong S.Y., Kim Y.H., Ma E.S., Woo M.H. (2011). A new cytotoxic coumarin, 7-[(*E*)-3′,7′-dimethyl-6′-oxo-2′,7′-octadienyl] oxy coumarin, from the leaves of Zanthoxylum schinifolium. Arch. Pharm. Res..

[92] Teponno R.B., Kusari S., Spiteller M. (2016). Recent advances in research on lignans, neolignans. Nat. Prod. Rep..

[93] Fuentealba C., Figuerola F., Estevez A.M., Bastias J.M., Munoz O. (2014). Bioaccessibility of lignans from flaxseed (Linum usitatissimum L.) determined by single-batch in vitro simulation of the digestive process. J. Sci. Food Agric..

[94] Zahir A., Ahmad W., Nadeem M., Giglioli-Guivarc’h N., Hano C., Abbasi B.H. (2018). In vitro cultures of Linum usitatissimum L.: Synergistic effects of mineral nutrients, photoperiod regimes on growth, biosynthesis of lignans, neolignans. J. Photochem. Photobiol. B.

[95] Gabr A.M.M., Mabrok H.B., Abdel-Rahim E.A., El-Bahr M.K., Smetanska I. (2017). Determination of lignans, phenolic acids, antioxidant capacity in transformed hairy root culture of Linum usitatissimum. Nat. Prod. Res..

[96] Schogor A.L.B., Huws S.A., Santos G.T.D., Scollan N.D., Hauck B.D., Winters A.L., Kim E.J., Petit H.V. (2014). Ruminal Prevotella spp. May Play an Important Role in the Conversion of Plant Lignans into Human Health Beneficial Antioxidants. PLoS ONE.

[97] Côrtes C., Gagnon N., Benchaar C., Da Silva D., Santos G.T.D., Petit H.V. (2008). In vitro metabolism of flax lignans by ruminal, faecal microbiota of dairy cows. J. Appl. Microbiol..

[98] Alvarez-Martinez F.J., Barrajon-Catalan E., Encinar J.A., Rodriguez-Diaz J.C., Micol V. (2018). Antimicrobial Capacity of Plant Polyphenols against Gram-positive Bacteria: A Comprehensive Review. Curr. Med. Chem..

[99] Nor Azman N.S., Hossan M.S., Nissapatorn V., Uthaipibull C., Prommana P., Jin K.T., Rahmatullah M., Mahboob T., Raju C.S., Jindal H.M. (2018). Anti-infective activities of 11 plants species used in traditional medicine in Malaysia. Exp. Parasitol..

[100] Chen P., Pang S., Yang N., Meng H., Liu J., Zhou N., Zhang M., Xu Z., Gao W., Chen B. (2013). Beneficial effects of schisandrin B on the cardiac function in mice model of myocardial infarction. PLoS ONE.

[101] Chun J.N., Cho M., So I., Jeon J.H. (2014). The protective effects of Schisandra chinensis fruit extract, its lignans against cardiovascular disease: A review of the molecular mechanisms. Fitoterapia.

[102] Olaru O.T., Niţulescu G.M., Orțan A., Dinu-Pîrvu C.E. (2015). Ethnomedicinal, Phytochemical, Pharmacological Profile of Anthriscus sylvestris as an Alternative Source for Anticancer Lignans. Molecules.

[103] Lin Y., Yngve A., Lagergren J., Lu Y. (2014). A dietary pattern rich in lignans quercetin, resveratrol decreases the risk of oesophageal cancer. Br. J. Nutr..

[104] Kyselka J., Rabiej D., Dragoun M., Kreps F., Burčová Z., Němečková I., Smolová J., Bjelková M., Szydłowska-Czerniak A., Schmidt S. (2017). Antioxidant, antimicrobial activity of linseed lignans, phenolic acids. Eur. Food Res. Technol..

[105] Vo Q.V., Nam P.C., Bay M.V., Thong N.M., Cuong N.D., Mechler A. (2018). Density functional theory study of the role of benzylic hydrogen atoms in the antioxidant properties of lignans. Sci. Rep..

[106] Sammartino A., Tommaselli G.A., Gargano V., di Carlo C., Attianese W., Nappi C. (2006). Short-term effects of a combination of isoflavones, lignans, Cimicifuga racemosa on climacteric-related symptoms in postmenopausal women: A double-blind, randomized, placebo-controlled trial. Gynecol. Endocrinol..

[107] McCann S.E., Thompson L.U., Nie J., Dorn J., Trevisan M., Shields P.G., Ambrosone C.B., Edge S.B., Li H.-F., Kasprzak C. (2010). Dietary lignan intakes in relation to survival among women with breast cancer: The Western New York Exposures, Breast Cancer (WEB) Study. Breast Cancer Res. Treat..

[108] Suzuki R., Rylander-Rudqvist T., Saji S., Bergkvist L., Adlercreutz H., Wolk A. (2008). Dietary lignans, postmenopausal breast cancer risk by oestrogen receptor status: A prospective cohort study of Swedish women. Br. J. Cancer.

[109] Cotterchio M., Boucher B.A., Kreiger N., Mills C.A., Thompson L.U. (2008). Dietary phytoestrogen intake—lignans, isoflavones—and breast cancer risk (Canada). Cancer Causes Control..

[110] Trock B.J., Hilakivi-Clarke L., Clarke R. (2006). Meta-analysis of soy intake, breast cancer risk. J. Natl. Cancer Inst..

[111] Lowcock E.C., Cotterchio M., Boucher B.A. (2013). Consumption of flaxseed, a rich source of lignans, is associated with reduced breast cancer risk. Cancer Causes Control..

[112] Buck K., Vrieling A., Zaineddin A.K., Becker S., Husing A., Kaaks R., Linseisen J., Flesch-Janys D., Chang-Claude J. (2011). Serum enterolactone, prognosis of postmenopausal breast cancer. J. Clin. Oncol..

[113] McCann S.E., Hootman K.C., Weaver A.M., Thompson L.U., Morrison C., Hwang H., Edge S.B., Ambrosone C.B., Horvath P.J., Kulkarni S.A. (2012). Dietary intakes of total, specific lignans are associated with clinical breast tumor characteristics. J. Nutr..

[114] Zaineddin A.K., Buck K., Vrieling A., Heinz J., Flesch-Janys D., Linseisen J., Chang-Claude J. (2012). The association between dietary lignans, phytoestrogen-rich foods, fiber intake, postmenopausal breast cancer risk: A German case-control study. Nutr. Cancer.

[115] Velentzis L.S., Cantwell M.M., Cardwell C., Keshtgar M.R., Leathem A.J., Woodside J.V. (2009). Lignans, breast cancer risk in pre-, post-menopausal women: Meta-analyses of observational studies. Br. J. Cancer.

[116] Buck K., Zaineddin A.K., Vrieling A., Linseisen J., Chang-Claude J. (2010). Meta-analyses of lignans, enterolignans in relation to breast cancer risk. Am. J. Clin. Nutr..

[117] Lin Y., Yngve A., Lagergren J., Lu Y. (2012). Dietary intake of lignans, risk of adenocarcinoma of the esophagus, gastroesophageal junction. Cancer Causes Control..

[118] Lin Y., Wolk A., Hakansson N., Lagergren J., Lu Y. (2013). Dietary intake of lignans, risk of esophageal, gastric adenocarcinoma: A cohort study in Sweden. Cancer Epidemiol. Biomarkers Prev..

[119] Zamora-Ros R., Guinó E., Alonso M.H., Vidal C., Barenys M., Soriano A., Moreno V. (2015). Dietary flavonoids, lignans, colorectal cancer prognosis. Sci. Rep..

[120] Wallstrom P., Drake I., Sonestedt E., Gullberg B., Bjartell A., Olsson H., Adlercreutz H., Tikkanen M.J., Wirfält E. (2018). Plasma enterolactone, risk of prostate cancer in middle-aged Swedish men. Eur J. Nutr..

[121] Eriksen A.K., Kyrø C., Nørskov N., Bolvig A.K., Christensen J., Tjønneland A., Overvad K., Landberg R., Olsen A. (2017). Prediagnostic enterolactone concentrations, mortality among Danish men diagnosed with prostate cancer. Eur. J. Clin. Nutr..

[122] Bylund A., Lundin E., Zhang J.X., Nordin A., Kaaks R., Stenman U.H., Aman P., Adlercreutz H., Nilsson T.K., Hallmans G. (2003). Randomised controlled short-term intervention pilot study on rye bran bread in prostate cancer. Eur. J. Cancer Prev..

[123] Hedelin M., Klint A., Chang E.T., Bellocco R., Johansson J.E., Andersson S.O., Heinonen S.M., Adlercreutz H., Adami H.O., Grönberg H. (2006). Dietary phytoestrogen, serum enterolactone, risk of prostate cancer: The cancer prostate Sweden study (Sweden). Cancer Causes Control..

[124] Anjum S., Abbasi B.H., Doussot J., Favre-Réguillon A., Hano C. (2017). Effects of photoperiod regimes, ultraviolet-C radiations on biosynthesis of industrially important lignans, neolignans in cell cultures of *Linum usitatissimum* L. (Flax). J. Photochem. Photobiol. B.

[125] Correa R.C.G., Peralta R.M., Haminiuk C.W.I., Maciel G.M., Bracht A., Ferreira I. (2018). New phytochemicals as potential human anti-aging compounds: Reality, promise, challenges. Crit. Rev. Food Sci. Nutr..

[126] Witkowska A.M., Waśkiewicz A., Zujko M.E., Szcześniewska D., Stepaniak U., Pająk A., Drygas W. (2018). Are Total, Individual Dietary Lignans Related to Cardiovascular Disease, Its Risk Factors in Postmenopausal Women? A Nationwide Study. Nutrients.

[127] Pellegrini N., Valtuena S., Ardigo D., Brighenti F., Franzini L., Del Rio D., Scazzina F., Piatti P.M., Zavaroni I. (2010). Intake of the plant lignans matairesinol, secoisolariciresinol, pinoresinol, lariciresinol in relation to vascular inflammation, endothelial dysfunction in middle age-elderly men, post-menopausal women living in Northern Italy. Nutr. Metab. Cardiovasc. Dis..

[128] Jacobs D.R., Pereira M.A., Meyer K.A., Kushi L.H. (2000). Fiber from whole grains, but not refined grains, is inversely associated with all-cause mortality in older women: The Iowa women’s health study. J. Am. Coll. Nutr..

[129] Vanharanta M., Voutilainen S., Rissanen T., Adlercreutz H., Salonen J.T. (2003). Risk of cardiovascular disease-related, all-cause death according to serum concentrations of enterolactone: Kuopio Ischaemic Heart Disease Risk Factor Study. Arch. Intern. Med..

[130] Franco O.H., Burger H., Lebrun C.E., Peeters P.H., Lamberts S., Grobbee D.E., Van Der Schouw Y.T. (2005). Higher dietary intake of lignans is associated with better cognitive performance in postmenopausal women. J. Nutr..

[131] Eichholzer M., Richard A., Nicastro H.L., Platz E.A., Linseisen J., Rohrmann S. (2014). Urinary lignans, inflammatory markers in the US National Health, Nutrition Examination Survey (NHANES) 1999–2004, 2005–2008. Cancer Causes Control..

